# Amyloidosis, Synucleinopathy, and Prion Encephalopathy in a Neuropathic Lysosomal Storage Disease: The CNS-Biomarker Potential of Peripheral Blood

**DOI:** 10.1371/journal.pone.0080142

**Published:** 2013-11-21

**Authors:** Bartholomew J. Naughton, F. Jason Duncan, Darren Murrey, Tierra Ware, Aaron Meadows, Douglas M. McCarty, Haiyan Fu

**Affiliations:** 1 Center for Gene Therapy, The Research Institute at Nationwide Children’s Hospital, Columbus, Ohio, United States of America; 2 Department of Pediatrics, The Ohio State University, Columbus, Ohio, United States of America; Justus-Liebig-University Giessen, Germany

## Abstract

Mucopolysaccharidosis (MPS) IIIB is a devastating neuropathic lysosomal storage disease with complex pathology. This study identifies molecular signatures in peripheral blood that may be relevant to MPS IIIB pathogenesis using a mouse model. Genome-wide gene expression microarrays on pooled RNAs showed dysregulation of 2,802 transcripts in blood from MPS IIIB mice, reflecting pathological complexity of MPS IIIB, encompassing virtually all previously reported and as yet unexplored disease aspects. Importantly, many of the dysregulated genes are reported to be tissue-specific. Further analyses of multiple genes linked to major pathways of neurodegeneration demonstrated a strong brain-blood correlation in amyloidosis and synucleinopathy in MPS IIIB. We also detected prion protein (Prnp) deposition in the CNS and Prnp dysregulation in the blood in MPS IIIB mice, suggesting the involvement of Prnp aggregation in neuropathology. Systemic delivery of trans-BBB-neurotropic rAAV9-hNAGLU vector mediated not only efficient restoration of functional *α-N*-acetylglucosaminidase and clearance of lysosomal storage pathology in the central nervous system (CNS) and periphery, but also the correction of impaired neurodegenerative molecular pathways in the brain and blood. Our data suggest that molecular changes in blood may reflect pathological status in the CNS and provide a useful tool for identifying potential CNS-specific biomarkers for MPS IIIB and possibly other neurological diseases.

## Introduction

Mucopolysaccharidosis (MPS) IIIB (OMIM #: 252920), is an autosomal recessive disorder caused by mutations in the gene that encodes α-N-acetylglucosaminidase (NAGLU), a lysosomal acid hydrolase that is essential in the stepwise degradation of heparan sulfate (HS) glycosaminoglycans (GAG) [1]. The resulting enzyme deficiency leads to a buildup of HS-GAGs within the lysosomes. The disease causing alleles of MPS IIIB are highly polymorphic, with over 80 mutations identified [2]. The lysosomal accumulation of HS-GAGs results in clinical disease, presenting predominantly severe progressive neurological disorders. Somatic manifestations of MPS IIIB do occur in all patients, but are mild, relative to other forms of MPS. Infants appear normal at birth but develop profound neurological manifestation at the age of 3–4 years, including reduced cognitive capacity, hyperactivity, seizures, and ultimately death [2].

The primary pathology of MPS IIIB has been characterized as the accumulation of HS-GAGs in lysosomes in cells of virtually all tissues/organs, especially in the CNS involving both neuronal and non-neuronal cells [1], [3]. While detailed mechanisms of pathology, especially neuropathology of MPS III, are not yet well understood, numerous studies have reported cascades of complex secondary pathological events in the CNS, including broad metabolic impairments [4], [5], neuroinflammation [5]–[10], oxidative stress [9], [11], autophagy [12], [13], and neurodegeneration [6], [8], [10], [14]–[19]. It is worth noting that many of these secondary neuropathological features of MPS IIIB, such as amyloid-*β* (Aβ) aggregation [14], [17], tauopathy [16], [17], synucleinopathy [15], [18], oxidative stress [9], [11] and neuroimflammation [5]–[10], are also common hallmarks of other neurodegenerative diseases like Alzheimer’s (AD) [20], [21] and Parkinson’s disease (PD) [22], [23]. In addition, our recent studies demonstrate widespread profound neuropathology in the peripheral nervous system (PNS) [24], indicating that neuropathological manifestation affects the entire nervous system.

No definite treatment is currently available for MPS IIIB and therapies have been limited to palliative treatment. The greatest challenge in developing therapies for MPS III has been the presence of the blood-brain-barrier (BBB). Significant therapeutic advancements have been made for treating lysosomal storage diseases (LSDs), such as hematopoietic stem cell transplantation (HSCT), recombinant enzyme replacement (ERT) and gene therapy [25]. MPS III (A–D) disorders are not amenable to currently approved systemic ERT or HSCT, which have shown somatic benefits, since the BBB precludes effective CNS access to either recombinant enzyme, or enzyme produced by transplanted hematopoetic stem cells. Alternative intrathecal ERT clinical trials are ongoing, targeting the CNS disorders in patients with MPS I, II and IIIA, and require repetitive administration on a regular basis. Gene therapy has been considered an ideal approach for treating LSDs because of the potential for long-term endogenous production of recombinant enzymes without the need to treat every cell. Numerous virus-mediated gene therapy studies, mostly designed to restore the missing enzyme activity, have shown various degrees of correction of lysosomal storage *in vitro* and *in vivo* in LSD animal models, using various viral vectors [26]. Recombinant adeno-associated viral (rAAV) has been a favored vector for gene delivery because it is non-pathogenic, with demonstrated long-term expression in the CNS and periphery [26], [27]. The recent finding of trans-BBB neurotropism of AAV9 offers an effective solution for CNS gene therapy, showing great potential for the treatment of LSDs and other neurological diseases [28]–[32]. A single systemic delivery of rAAV9 vectors can lead to global CNS and widespread somatic restoration of enzyme activity, correction of lysosomal storage pathology, and functional neurological benefits in mice with MPS IIIB or IIIA [28], [29].

As therapeutic development advances and more therapies for MPS become available, the lack of biomarkers will be a critical challenge for therapeutic assessment. Urinary GAGs and specific lysosomal enzymes have been the only biomarkers for MPS, although recent studies identified serum heparin cofactor II-thrombin (HCII-T) as a biomarker for MPS I, II and III [33], dipeptidyl peptidase IV (DPP-IV) for MPS I, II, III, IVA and VI [34], and recently the potential of disease-specific non-reducing end carbohydrate biomarkers for MPS [35]. However, there are currently no specific biomarkers for MPS corresponding to neurological disease severity or therapeutic responsiveness. The critical challenge for finding CNS-specific biomarkers stems from limited access to the CNS tissues of patients, especially at early disease stages.

The demonstrated neuroinflammation in MPS IIIB [10] led to our hypothesis of potential links between the CNS and periphery, likely through immune modulation. Using gene expression analyses, this study demonstrates for the first time a strong correlation between the brain and peripheral blood cells in neurodegeneration in a mouse model of MPS IIIB. Notably, a single systemic delivery of rAAV9-hNAGLU vector led to the correction of multiple dysregulated neurodegeneration-associated genes in both the brain and blood, strongly supporting the CNS biomarker potential of peripheral blood for MPS IIIB.

## Methods

### Animals

A NAGLU knockout (MPS IIIB) mouse colony [3] was maintained on an inbred background (C57BL/6) of backcrosses from heterozygotes in the vivarium at NCH-RI. All animal care and procedures were in accordance with the Guide for the Care and Use of Laboratory Animals and the protocol that was approved by the Institutional Animal Care and Use Committee at the Research Institute at Nationwide Children’s Hospital. The genotypes of progeny mice were identified by PCR.

MPS IIIB mice and their age-matched wildtype (wt) littermates were used in the experiments. To avoid sex-related variations, only male mice were used in a majority of assays, with the exception of gene expression microarrays that used pooled RNA samples from both male and female mice.

### Injection of Recombinant AAV9 (rAAV9) Viral Vector

A previously described conventional single-strand rAAV vector plasmid was used to produce rAAV9-CMV-hNAGLU viral vector [28], [36]. The vector genome contained AAV2 terminal repeats, a CMV immediate-early promoter, SV40 splice donor/acceptor signals, a human NAGLU coding sequence and polyadenylation signal from the bovine growth hormone gene. The viral vectors were produced in HEK293 cells using three-plasmid co-transfection including AAV helper plasmid encoding serotype 9 capsid, and purified following published procedures [37].

MPS IIIB mice, 4–6-week old (n = 30, M:F = 1∶1), were anesthetized by an intraperitoneal injection of 2.5% Avertin (0.3–0.4 µg/g body weight) prior to an intravenous injection of rAAV9 vector (1×10^13^ vector genome/kg, in 150–200 µl PBS) via tail vein.

### Behavioral Tests: Hidden Task in the Morris Water Maze

The rAAV9-CMV-hNaGlu-treated MPS IIIB mice (n = 13, M:F = 7∶6) and controls [wt:n = 25 (M:F = 16∶13; non-treated MPS IIIB: n = 39 (M:F = 20∶19)] were tested for behavioral performance at approximately 5.0–5.5 months of age in a Morris water maze. [38] The water maze consisted of a large circular pool (diameter = 122 cm) filled with water (45 cm deep, 24–26°C) containing 1% white TEMPERA paint, located in a room with numerous visual cues. Mice were tested for their ability to find a hidden escape platform (20×20 cm) 0.5 cm under the water surface. Each animal was given four trials per day, across 4 days. For each trial, the mouse was placed in the pool at one of four randomly ordered locations, and then given 60 seconds (sec) to swim to the hidden escape platform. If the mouse found the platform, the trial ended, and the animal was allowed to remain 10 sec on the platform before the next trial began. If the platform was not found, the mouse was placed on the platform for 10 sec, and then given the next trial. Measures were taken of latency to find the platform, swimming distance (cm), and swimming velocity (cm/min) through an automated tracking system (San Diego Instruments, San Diego, CA, USA).

### Longevity Assessment

Following the rAAV9-hNaGlu vector injection(s), mice were continuously observed for the development of endpoint symptoms, or until death occurred. The endpoint was when the symptoms of late stage clinical manifestation (urine retention, rectal prolapse, protruding penis) in MPS IIIB mice became irreversible, or when mice showed significant weight loss, dehydration or morbidity.

### Tissue Analyses

Tissue analyses were carried out when mice were 2 or 6 months of age (n = 9–12/group). The mice were anesthetized with an IP injection of 2.5% Avertin and perfused transcardially with cold PBS (0.1 M, pH 7.4), prior to blood collection by cardiac puncture and tissue collection. Each mouse brain was divided into 2 spheres along the middle line. Half brain and somatic tissues were collected on dry ice and stored at −80°C before being processed for analyses. The remaining half brain was kept in 4% paraformaldehyde overnight for paraffin sectioning (4 µm).

#### NAGLU activity assay

Tissue samples were assayed for NAGLU enzyme activity following published procedure with modification [3], [36], [39]. The assay measures 4-methylumbelliferone, a fluorescent product formed by hydrolysis of the substrate 4-methylumbelliferyl-N-acetyl-α-Dglucosaminide. The NAGLU activity is expressed as unit/mg protein. One unit is equal to 1 nmol 4-methylumbelliferone released/hour at 37°C.

#### GAG content measurement

GAG was extracted from tissues following published procedures with modification [40], [41]. Dimethylmethylene blue (DMB) assay was used to measure GAG content [42]. The GAG samples (from 0.5 to 1.0 mg tissue) were mixed with H2O to 40 µl before adding 35 nM DMB (Polysciences) in 0.2 mmol/l sodium formate buffer (pH 3.5). The product was measured using a spectrophotometer (OD_535_). The GAG content was expressed as µg/mg tissue.

#### Total RNA isolation

Paxgene Blood RNA Kit (Qiagen) was used to extrat total RNAs from whole blood, and Qiagen RNeasy Kit (Qiagen) was used to isolate total RNAs from tissue samples, following the protocols recommended by the manufacturer.

#### Gene expression microarray analysis

Gene expression profiling was performed following manufacturer recommended procedures on pooled total RNA samples from 6-month-old wt or MPS IIIB mice (n = 6/group, m:f = 1∶1), using 430 2.0 GeneChips® (Affymetrix Inc.), containing probe sets that measure 39,000 transcripts from mouse RNA. The probes were based on the gene sequences available in GenBank®, dbEST, and RefSeq. GeneChips were scanned in a GeneChip Scanner 3000 (Affymetrix Inc.). CEL files were generated from DAT files using GeneChip® Operating Software (GCOS, Affymetrix Inc.). The probe set signals were generated using the RMA algorithm in ArrayAssist 3.4 (Stratagene) and are used to determine differential gene expression by pair-wise comparisons. The fold changes (FC) of gene expression in MPS IIIB *vs*. wt mice is used for further interpretation of the microarray data. Functional analyses of array data were performed using Ingenuity Pathway Analysis (IPA) software and Database for Annotation, Visualization and Integrated Discovery (DAVID).

#### Real-time qRT-PCR

Real-time qRT-PCR for the genes of interest derived from gene expression microarray data, was performed using total RNA from individual mice as templates (n = 9–12). The primer and probe sequences are listed in Supplementary Table S1. A pair of primers for murine β-actin gene was used as internal control. Reverse transcriptase reactions were performed using the Superscript III First Strand Synthesis Kit (Life Technologies). Real-time qPCR reactions were performed using Absolute Blue QPCR Mix (Thermo Scientific, Waltham, MA) and Applied Biosystems 7000 Real-Time PCR System (Life technologies, Carlsbad, CA), following the procedures recommended by the manufacturer. All reactions were run in triplicate as a duplex reaction, with probes conjugated to either Fam or Hex (Sigma, St Louis, MO). The levels of tested mRNAs relative to β-actin mRNA were determined, and standard curves were applied [43]. Data were expressed as relative quantitation of gene expression, in MPS IIIB and rAAV9-treated MPS IIIB mice vs. wt.

#### Immunohistochemistry (IHC)

Immunohistochemistry was performed on paraffin sections (4 µm) using antibodies against Necab3 (Proteintech), α-synuclein (Abcam), Apbb2 (LSBio), parkin (LSBio), and Prion Protein (Origene) GFAP (Millipore, Billerica, MA), LAMP-1 (Novus) and hNAGLU [28] with corresponding secondary antibodies conjugated with HRP (Sigma, St. Louis, Mo) or AlexaFluo (Invitrogen). The IHC sections were visualized under a light microscope. All settings images (such as exposure, aperture and illumination) were kept constant for image acquisition. Relative percent optical density (staining area) was quantified from a standardized size box in each region, using ImageJ software (NIH, Bethesda, MD). For every brain area investigated, three images were taken from each of three different sections. Once analyzed, these were averaged into a single value, which was used to represent that brain area in each mouse (n = 7/group), which was used for statistical analysis. Sections stained by immunofluorescence were visualized under a fluorescent microscope.

#### ELISA

Brain whole cell proteins were extracted from cortical tissue of each animal (n = 9–12) using RIPA buffer containing HALT Protease and Phosphatase Inhibitor Cocktail (Thermo Scientific). The brain protein samples were assayed by ELISA for phosphorylated amyloid beta (A4) precursor protein (APP), using a primary antibody against APP phosphorylated at Thr^668^ (R&D Systems) and a secondary antibody conjugated with horse radish peroxidase (Sigma), following the protocols recommended by the manufacturers. The results (OD_45_) were obtained using a SpectraMax M2 microplate reader (Molecular Devices, Sunnyvale, CA).

### Statistics

Data were analyzed using student t-test and/or separate one-way ANOVAs to examine group differences. For all comparisons, significance was set at P<0.05.

## Results

### Lysosomal Storage of HS-GAG Leads to Broad Blood Gene Dysregulation in MPS IIIB Mice, Including Impaired Expression of Tissue-specific Genes

**Figure 1:**
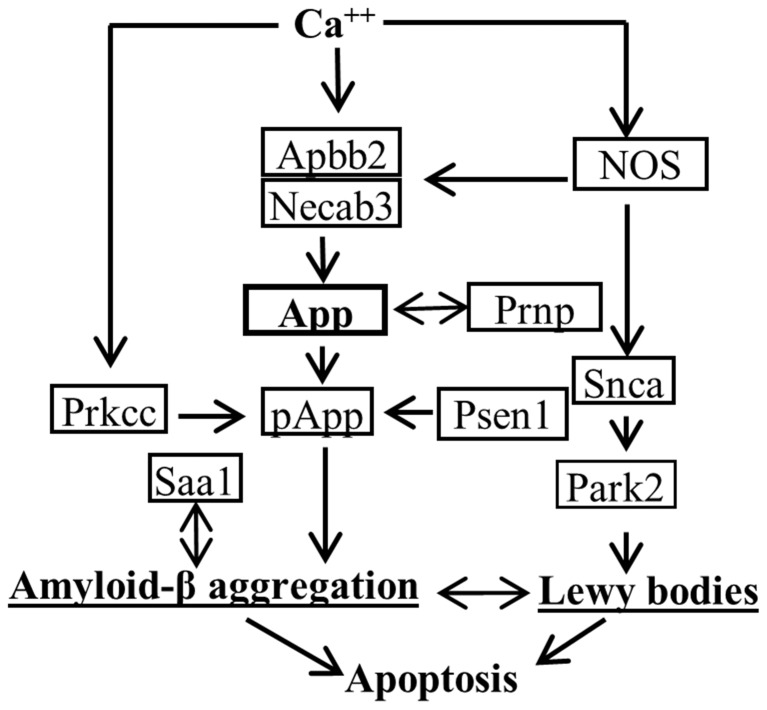
Illustration of major molecular components of pathways linked to neurodegeneration.

In this study, we performed genome-wide gene expression microarray (39,000 transcripts) on pooled brain and blood RNA samples from 6-month-old MPS IIIB mice and wt littermates (n = 6/group, m:f = 1∶1), to identify molecular signatures in peripheral blood that are possibly relevant to MPS IIIB neuropathology. The results showed significant dysregulation (>2 fold) of 2,802 genes in blood, and 281 genes in the brain in MPS IIIB mice, compared to wt (**Table 1**). Functional analyses using IPA software and DAVID showed that the dysregulated genes are known to be involved in a broad range of biological processes, involving virtually all tissues, albeit some with strong tissue association with brain, liver, or lung expression (**Table** **1**). Furthermore, the blood gene dysregulation broadly reflects the complexity of MPS IIIB pathology, involving virtually all known aspects of the disease, including inflammation, neurodegeneration, autophagy, oxidative stress and metabolic impairments (GAGs, lipid, fatty acid, cholesterol metabolism), DNA and cellular repair processes, as well as previously unexplored potential pathological components of the disease (**Table 1**). **Table 2** lists some of the genes dysregulated in blood which are known to be major elements of pathways linked to other neurodegenerative diseases, such as Alzheimer’s (AD) and/or Parkinson’s disease (PD), as illustrated in **Fig. 1**. While the array was performed using pooled RNA samples, these data suggest that gene dysregulation in blood may be associated with neuropathology in the CNS.

**Table 1:** Functional and disease association of blood transcriptional profile in MPS IIIB mice.

Function/disease association	Blood genes (FC>2)			Brain Genes (FC>2)	
	No. genes	%**		No. genes	%**
**Total**	2,802	–		281	–
Unknown Genes	575	–		43	–
Known Genes	2,227	–		238	–
Neurological function & neurological Diseases	550	24.7		56	23.5
Alzheimer’s Disease	121	5.4		6	2.5
Parkinson’s Disease	33	1.5		6	2.5
Inflammation	197	8.9		68	24.2
Metabolism	319	14.3		32	13.5
Cell death and survival	90	4.9		62	26.1
Tissue association*					
Brain	144	9.7		26	10.9
Liver	729	32.7		47	19.8
Lung	172	7.7		18	7.6

Genome-wide gene expression microarrays were performed on pooled blood and brain RNA samples from 6 mo-old MPS IIIB mice and their wt littermates (n = 6/group, M:F = 1∶1). Genes of transcripts were identified by Ingenuity Pathway Analysis (IPA).

* Genes associated with specific tissues according to enrichment analysis using Database for Annotation, Visualization and Integrated Discovery (DAVID); **FC>2:** fold change >2;

** % of known genes.

**Table 2:** Dysregulation of neurodegeneration associated genes in 6-month-old MPS IIIB mice.

Gene symbol	Gene name	GeneBank access #	Fold of change*	
			Blood	Brain
Apbb2	Amyloid beta (A4) precursor protein-binding, family B, member 2	NM_001201413	+1.85	+1.33
Necab3	N-terminal EF-hand calcium binding protein 3	NC_021546	−2.45	+1.19
App	Amyloid beta (A4) precursor protein	NM_001198825	−2.57	+1.22
Saa1	Serum amyloid A 1	NM_009117	+9.09	−2.04
Snca	Synuclein, alpha	NC_009221	−1.90	+1.45
Psen1	Presenilin 1	NC_008943	−2.26	−1.41
Prkcg	Protein kinase C, gamma	NC_011102	−3.02	−1.54
Park2	Parkinson disease (autosomal recessive, juvenile) 2, parkin	NC_016694	−1.92	+1.25
Prnp	Prion protein	NC_011170	+2.50	+1.07
Bace2	beta-site APP-cleaving enzyme 2	NC_019517	+4.17	+1.15
Htr2B	5-hydroxytryptamine (serotonin) receptor 2B	NC_008311	+4.79	+1.01

*Gene expression microarray data were expressed as fold changes in MPS IIIB mice, relative to wt.

+increase;

−decrease.

### Temporal Association between Blood Gene Dysregulation and Neurodegeneration in MPS IIIB

Because gene microarrays used pooled RNA samples, qRT-PCR was performed to confirm the reliability of array data using RNA samples from individual animals. To further investigate the biomarker potential of blood for neuropathology, individual samples from 2- or 6-month-old MPS IIIB and wt mice (n = 9–12/group) were analyzed for the expression of multiple genes in peripheral blood and brain by qRT-PCR, ELISA and IHC. These genes (**Table 2**) were selected as targets because they were shown by array to be dysregulated in blood and/or brain, and are known to be involved in neurodegenerative processes, such as amyloid-β (Aβ) deposition and protein aggregation in the brain.

#### Early emergence of molecular impairments linked to amyloidosis in the CNS was reflected in peripheral blood

**Figure 2:**
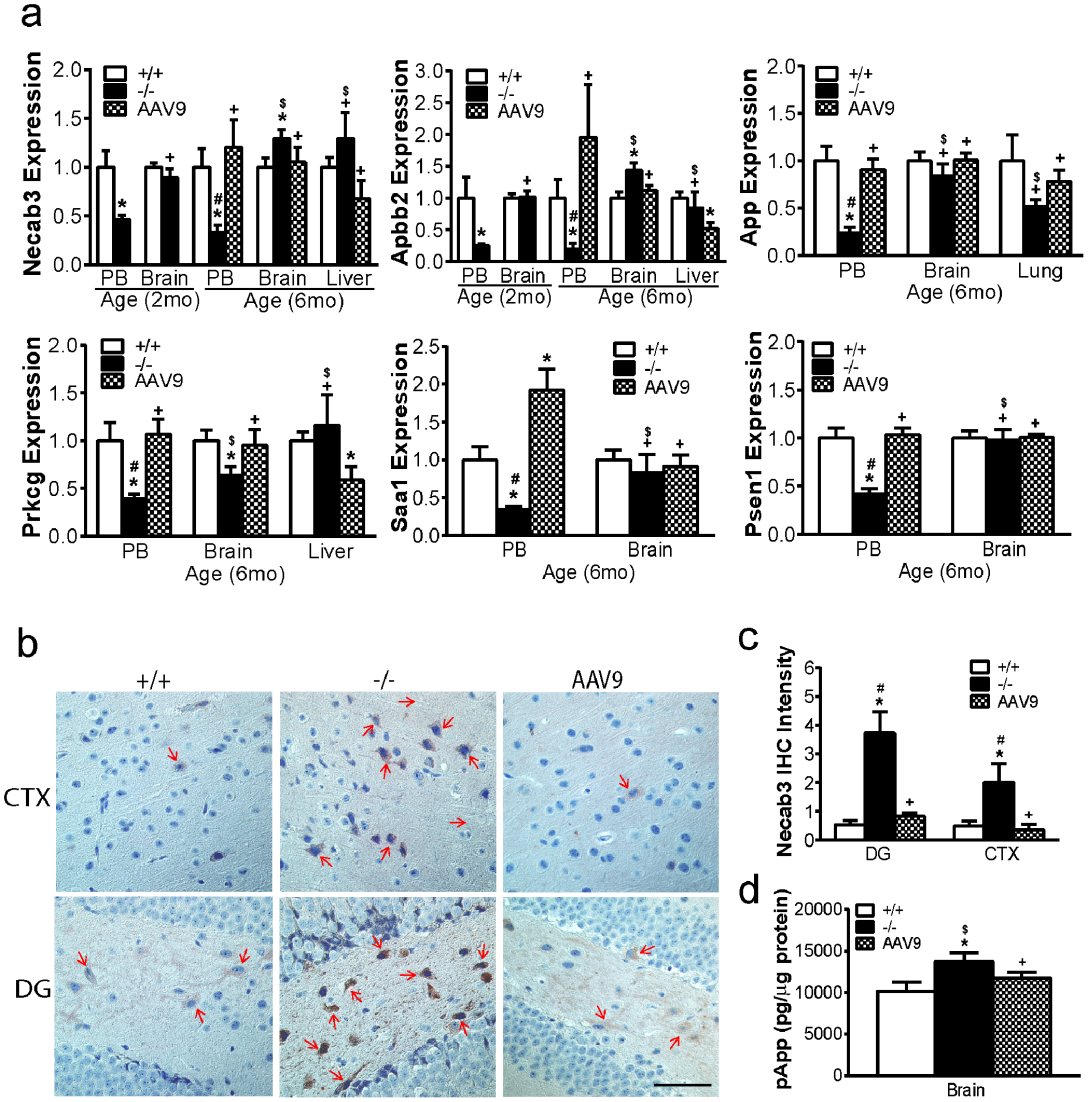
Dysregulation of pathways involved in amyloidosis in the brain and blood in MPS IIIB mice and their response to rAAV9-hNAGLU gene delivery. **a.** Total RNA from the brain (cortex), peripheral blood (PB) and/or representative control somatic tissues of 2 mo and/or 6 mo old wt, MPS IIIB mice, and MPS IIIB mice treated with an IV injection of rAAV9-CMV-hNAGLU (1×10^13^ vg/kg), were assayed by qRT-PCR (n = 9–12/group). Data: relative expression vs. wt. **b & c.** Brain tissue sections (4 µm) from 6 mo-old mice (n = 7) were assayed by IHC for Necab3. Necab3-positive calls/signals were stained brown (**b**) (red arrows). Necab3 staining intensity was quantitated using ImageJ (**c**). **d.** Whole cell proteins of brain cortical tissues from 6 mo-old mice were assayed by ELISA for pApp levels. **+/+:** wt mice; **−/−:** non-treated MPS IIIB mice; **AAV9:** rAAV9-treated MPS IIIB mice; CTX: cerebral cortex; DG: dentate gyrus of hippocampus. *: P<0.05 vs. wt; **#:** P<0.05 vs. AAV9-treated; **+:** P>0.05 vs. wt; **$:** P>0.05 vs.AAV9-treated. Scale bar: 50 µm.

To assess the gene expression aberrations involved in Aβ deposition, we analyzed changes in Necab3, Apbb2, App, Saa1, Psen1 and Prkcg. qRT-PCR showed a significant reduction in the expression of Necab3 and Apbb2 in blood in both 2 mo- and 6 mo-old MPS IIIB mice compared with wt (**Fig. 2a**). The blood expression of App, Saa1, Prkcg and Psen1 were significantly decreased only in 6 mo-old MPS IIIB mice. While no changes in the expression levels of these genes were detected in the brain in MPS IIIB mice at 2 mo of age, we observed significant increases in the transcripts of Necab3 and Apbb2, but reduced expression of Prkcg, in the brain at 6 mo of age (**Fig. 2a**). qRT-PCR did not detect significant transcript changes in App, Saa1 and Psen1 in the brain. Importantly, our data showed no significant changes in the expression of any of these genes in representative control somatic tissue (liver or lung) from MPS IIIB mice compared to wt (**Fig. 2a**). The qRT-PCR results in the brain were confirmed using IHC, demonstrating a significant increase in Necab3 protein in the brain of 6 mo-old MPS IIIB mice (**Fig. 2b, 2c**). The accumulation of Necab3 appeared to be intracellular in neurons, and was observed throughout the brain, predominantly in the cortex and hippocampus (**Fig. 2b, 2c**) as well as sporadically in the thalamus, striatum and brain stem (data not shown). Furthermore, while qRT-PCR showed no significant change in App at the mRNA level in the brain (**Fig. 2a**), using ELISA, we detected a significant increase in phosphorylated App (pApp), the suspected pathogenic form, in the cortical brain tissues of 6 mo-old MPS IIIB mice (**Fig. 2d**). In contrast, qRT-PCR did not support alteration in Bace2 and Htr2B in either brain or blood, as reported in the gene arrays.

These data suggested that the dysregulation of molecular pathways linked to Aβ deposition was likely associated with amyloidosis in the CNS during the progression of MPS IIIB, and that these changes were reflected in blood. Our results also indicate an early emergence of neurodegeneration that may be detectable in peripheral blood at an early stage of the disease, when changes in the brain are too low to be detected, supporting the concept of blood as a potential biomarker for MPS IIIB CNS pathology.

#### Dysregulation in genes associated with synucleinopathy in the CNS and blood

**Figure 3:**
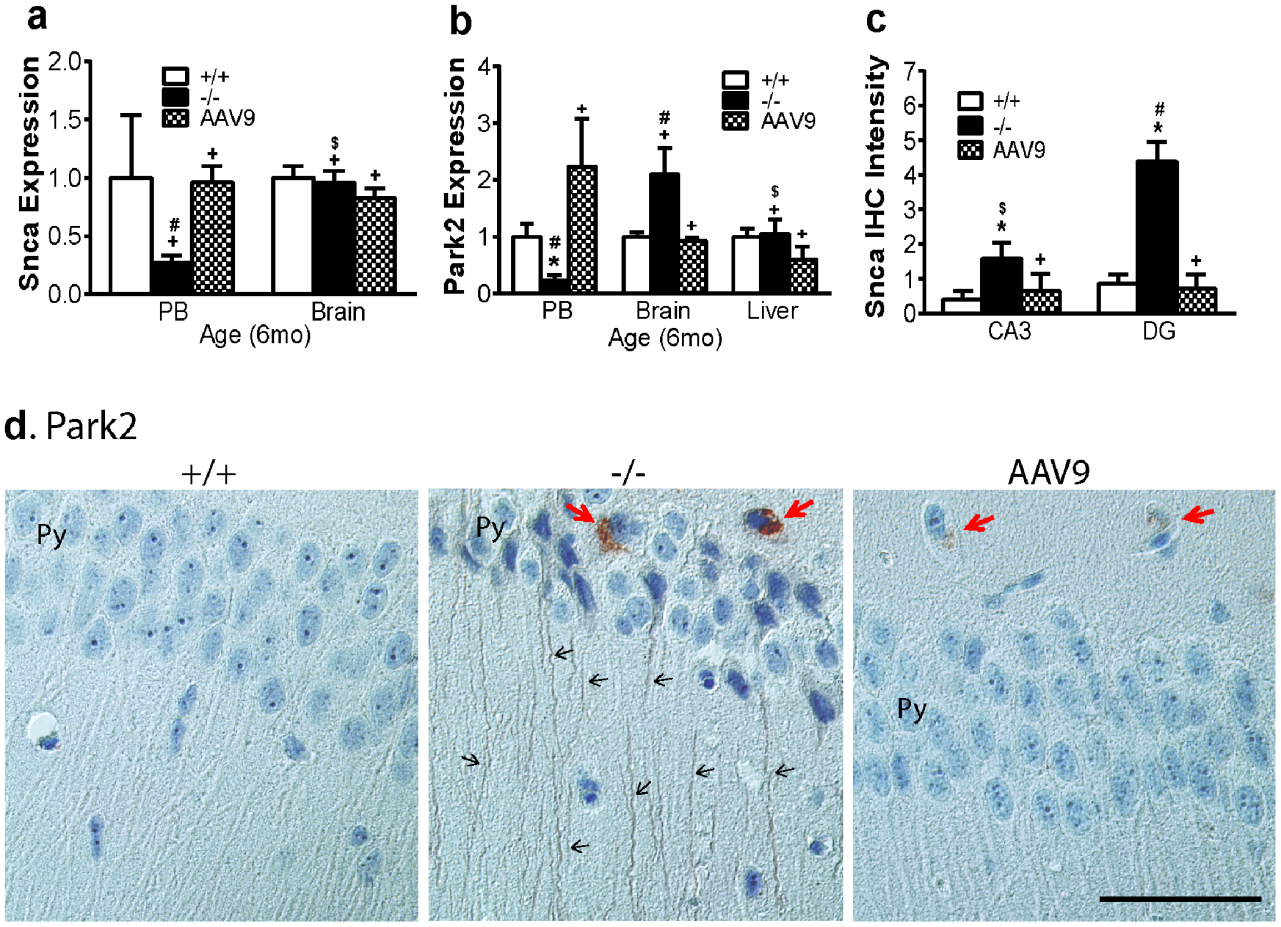
Gene dysregulation involved in synucleinopathy in the brain and blood in MPS IIIB mice and their response to rAAV9-hNAGLU gene delivery. **a & b**: Total RNA from the brain (cortex), peripheral blood (PB) and control somatic tissues of 6 mo old mice were assayed for Snca (**a**) and Park2 (**b**) by qRT-PCR (n = 9–12/group). Data: relative expression vs. wt. Brain tissue sections (4 µm) from 6 mo-old mice (n = 7) were assayed by IHC for Snca (**c**) and Park2 (**d**), positive cells/signals were stained brown (d). Snca staining intensity was quantitated using ImageJ (**c**). **+/+:** wt mice; **−/−:** non-treated MPS IIIB mice; **AAV9:** rAAV9-treated MPS IIIB mice; **CA3:** hippocampus CA3 region; **DG:** dentate gyrus; **Py:** pyramidal cell layer of hippocampus; **red arrows:** Park2-positive neuronal cell bodies; **black arrows:** Park2-positive neuronal processes. *: P<0.05 vs. wt; **#:** P<0.05 vs. AAV9-treated; **+:** P>0.05 vs. wt; **$:** P>0.05 vs.AAV9-treated. Scale bar: 50 µm.

Using qRT-PCR, we observed significant decreases in the transcription of both Snca and Park2 in blood, and significantly increased expression of Park2, but not Snca, in the brain in 6 mo-old MPS IIIB mice (**Fig. 3a, 3b**). We did not detect significant changes in Snca or Park2 expression in liver tissue, though it does exhibit extensive lysosomal storage pathology. There were no detectable changes in Snca or Park2 expression in either blood or the brain of MPS IIIB mice at 2 mo of age. Increases in both Snca (**Fig. 3c**) and Park2 (**Fig. 3d**) proteins were visible by IHC in the brains of 6 mo-old MPS IIIB mice, predominantly in hippocampus**,** the laminae I and II of the spinal cord dorsal horn, and/or cerebral cortex (Park2) or granule layer of cerebellum (Snca). Snca deposition appeared to be intra-axonal or extracellular (data not shown), while the accumulation of Park2 was intracellular in the cytoplasm and processes of neurons (**Fig. 3d**). These results support the involvement of synucleinopathy and Park2 deposition in the neuropathology of MPS IIIB, with a strong correlation to the dysregulation of these genes in blood. These data further support the potential role of blood cells in neurodegeneration and the CNS-biomarker potential of blood for MPS IIIB.

#### Prionopathy and brain-blood association

**Figure 4:**
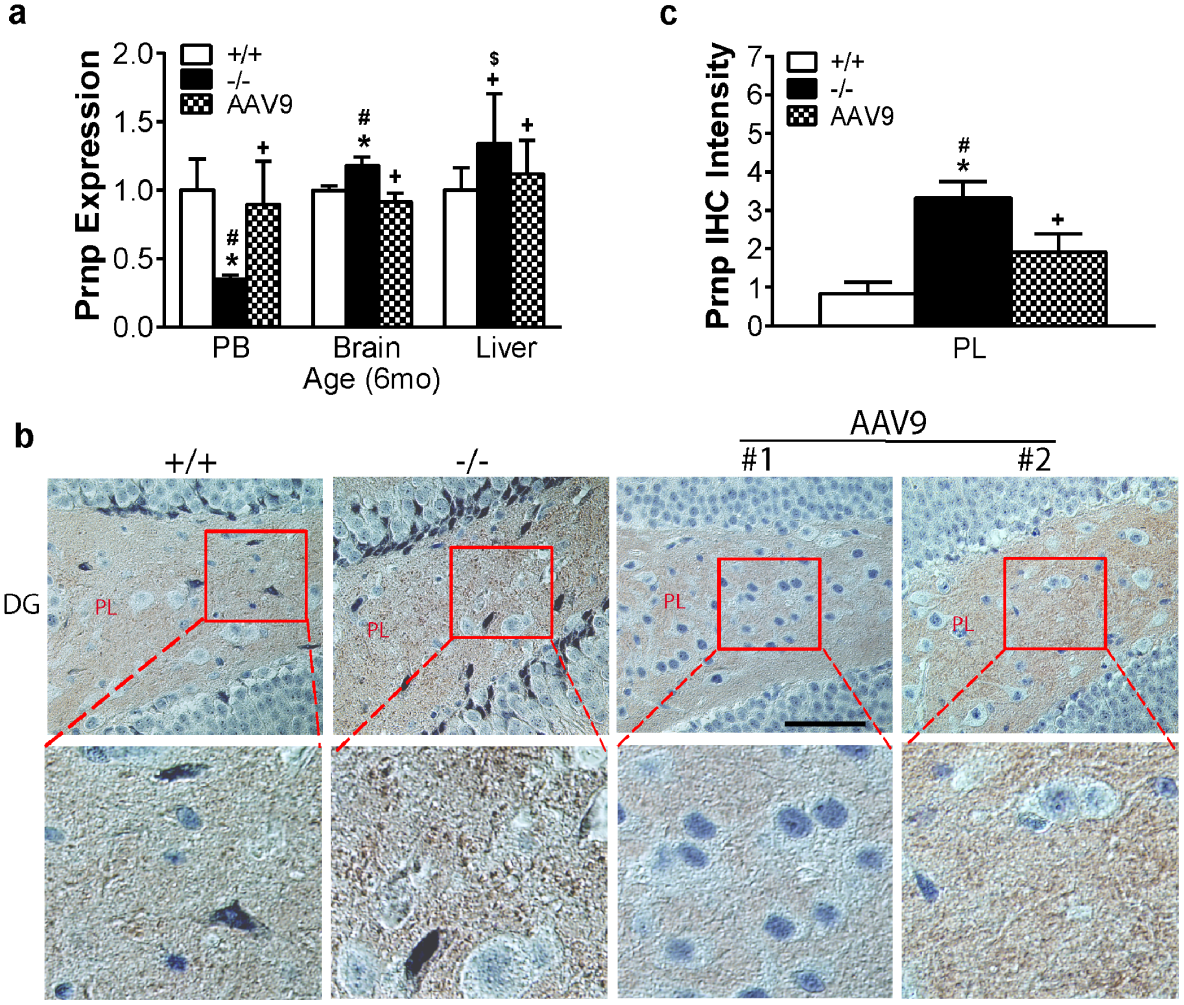
Dysregulation in prion protein in the brain and blood in MPS IIIB mice and their response to rAAV9-hNAGLU gene delivery. Total RNA from the brain (cortex), peripheral blood (PB) and control somatic tissues of 6 mo old mice were assayed for Prnp by qRT-PCR (n = 9–12/group) (**a**). Data: relative expression vs. wt. Brain tissue sections (4 µm) from 6 mo-old mice (n = 7) were assayed for Prnp by immunohistochemistry (**b, c**). Prnp-positive signals were stained brown (**b**). Prnp staining intensity in PL was quantitated using ImageJ (**c**). **+/+:** wt mice; **−/−:** non-treated MPS IIIB mice; **AAV9:** rAAV9-treated MPS IIIB mice; **#1:** mouse with low Prnp IHC intensity; **#2:** mouse with high Prnp IHC intensity; **DG:** dentate gyrus of hippocampus. **PL:** polymoph layer of DG; *: P<0.05 vs. wt; **#:** P<0.05 vs. AAV9-treated; **+:** P>0.05 vs. wt; **$:** P>0.05 vs.AAV9-treated. Scale bar: 50 µm.

Analysis by qRT-PCR showed significant up-regulation of Prnp in the brain and down-regulation in peripheral blood in MPS IIIB mice at 6 mo (**Fig. 4a**), but not at 2 mo of age. We did not observe significant change in Prnp transcript level in representative somatic tissue (**Fig. 4a**). Immunohistochemistry showed significantly increased Prnp protein (**Fig. 4b, 4c**) in the brain of 6 mo-old MPS IIIB mice, compared to wt. The Prnp deposition appeared to be intra-axonal or extracellular, predominantly in the hippocampus (**Fig. 4b**), laminae I and II of the spinal cord dorsal horn, and cerebral cortex (data not shown). These results indicate the presence of Prnp aggregation in the CNS during the disease progression in MPS IIIB mice, likely a component of neurodegeneration. Importantly, this too correlated with changes in blood.

### Therapeutic Gene Delivery to Test for Normalization of Aberrant Transcription in Peripheral Blood and Brain

To determine the CNS therapeutic surrogate potential of blood gene profiles for MPS IIIB, we treated 4–6 wk old MPS IIIB mice (n = 30) with a single intravenous injection of rAAV9-CMV-hNAGLU vector (1×10^13^ vg/kg). Serotype 9 rAAV vectors cross the BBB and a systemic rAAV9-CMV-hNAGLU delivery was previously shown to result in both CNS and somatic correction of pathology [28].

**Figure 5:**
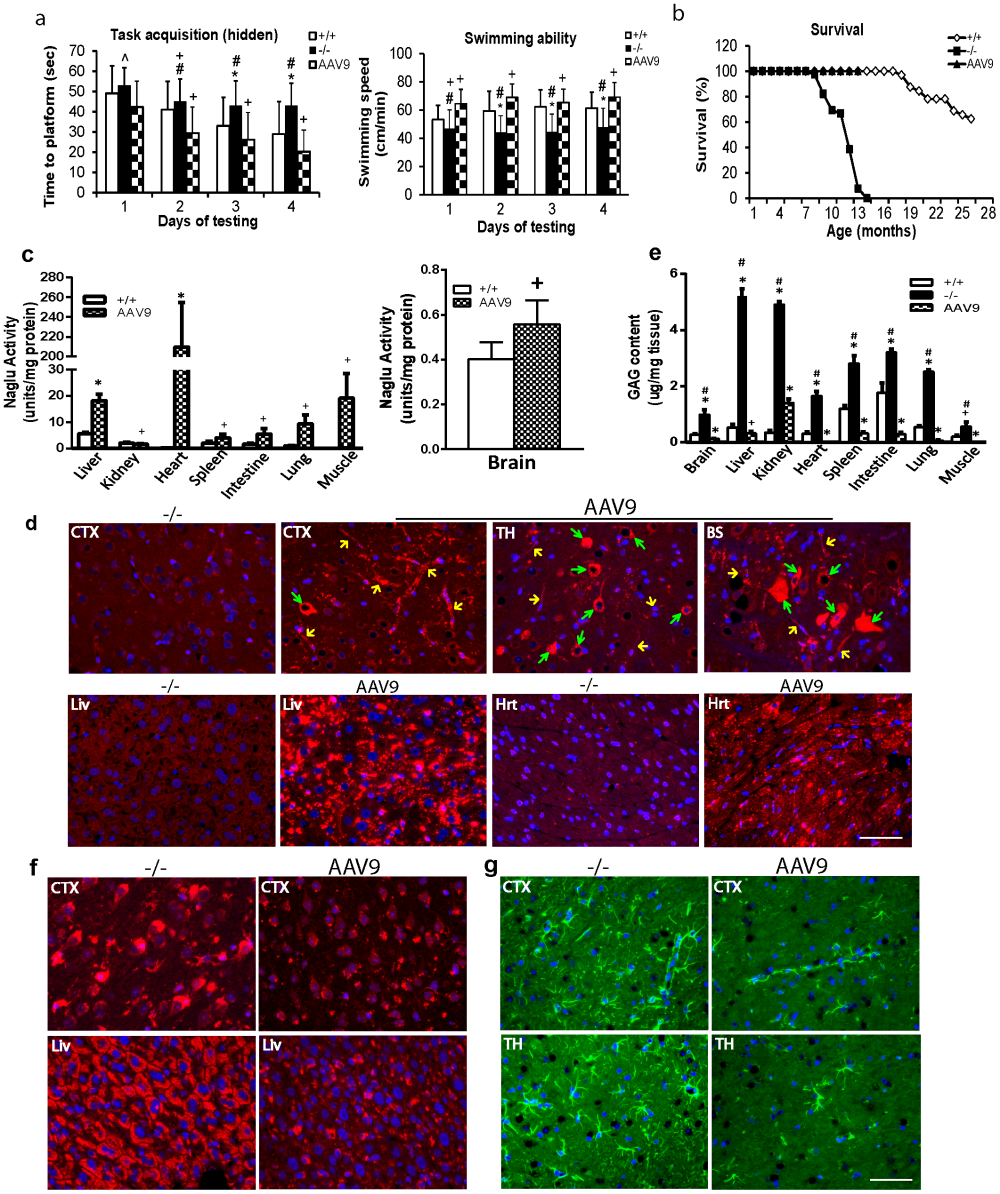
rAAV9-mediated restoration of NAGLU activity, correction of GAG storage and astrocytosis, and functional benefits MPS IIIB mice. 4–6wk old MPS IIIB mice were treated with an IV injection of rAAV9-CMV-hNAGLU vector (1×10^13^ vg/kg). Mice were tested for behavior in Morris water maze at 5–5.5 mo old (n = 13) (**a**). Longevity studies are ongoing (n = 11) (**b**).Tissue analyses were performed at 6 mo of age. **c.** tissue NAGLU activity (no detectable NAGLU activity in tissues from non-treated MPS IIIB mice). **d.** Immunofluorescence (IF) for hNAGLU. Red fluorescence: hNAGLU-positive cells/signals. CTX: cerebral cortex; TH: thalamus; **BS:** brain stem; **Live:** liver; **Hrt:** heart. **Green arrows:** hNAGLU-positive neurons; **Yellow arrows:** hNAGLU positive vasculatures. **e.** tissue GAG contents; **f.** IF staining for LAMP-1. Red fluorescence: LAMP-1-positive calls/signals. g. IF staining for GFAP. Green fluorescence: GFAP-positive cells/signals. *: P<0.05 vs. WT; **#:** P<0.05 vs. AAV9-treated; **+:** P>0.05 vs. WT; ^∧^: P>0.05 vs +/+ and AAV9-treated. Scale bar: 50 µm.

The animals were tested for behavior in a hidden task in Morris water maze when they were 5–5.5 mo old (n = 13, M:F = 7/6). Similar to our previously published data, rAAV9-treated MPS IIIB mice exhibited significant improvement in latency to find a hidden platform and swimming ability in water maze (**Fig. 5a**), indicating the correction of neurological disorders. Longevity studies in 11 rAAV9-treated animals are currently ongoing, and all of them have lived >15 mo (**Fig. 5b**). Non-treated MPS IIIB mice died at 7.2–13.5 mo of age (**Fig. 5b**). These data further support the functional benefits of the rAAV9 gene delivery, since premature death in MPS IIIB has been attributed to neurological disorders.

Blood and tissue samples from male mice were analyzed at 6 mo (n = 8) of age to assess the therapeutic impacts of rAAV9 gene delivery on the restoration of tissue NAGLU activity, and the correction of lysosomal storage pathology and neuropathology.

Tissues were assayed for NAGLU enzymatic activity to quantify the expression and functionality of the transgene. Similar to our previous observation, [28] NAGLU activity was detected at wt levels in the brain. We also detected NAGLU activity at normal or subnormal levels in the lung and intestine (**Fig. 5c**), supra-physiologic levels in the liver, skeletal muscles (**Fig. 5c**) and heart, and low levels in the spleen and kidney (**Fig. 5c**). No detectable NAGLU activity (<0.03 unit/mg) was observed in tissues from non-treated MPS IIIB mice. Using immunofluorescence, hNAGLU-specific signals were detected throughout the brains of treated mice, in neurons, glia (likely oligodendrocytes by morphology), and abundant endothelial cells in capillaries and larger blood vessels (**Fig. 5d**). The rNAGLU protein was detected in >50% of hepatocytes, >95% of cardiomyocytes (**Fig. 5d**) and 10–30% of skeletal myocytes (data not shown). The distribution of rAAV9-transduced hepatocytes was uniform throughout the liver. We also observed transduction in abundant neurons in dorsal root ganglion (DRG) and intestinal neurons in myenteric plexus and submucosal plexus (data not shown), indicating the peripheral nervous system (PNS) targeting. The rNAGLU signals were mostly present in granules (**Fig. 5d** **)**, suggesting correct lysosomal trafficking of rNAGLU.

Tissues were also assayed for GAG content to quantify the impact of rAAV9-NAGLU gene delivery on the lysosomal storage pathology in MPS IIIB mice. The treatment resulted in a reduction of GAG content to normal levels in the brain, liver, heart, lung, intestine, spleen and skeletal muscle, but with partial GAG reduction in kidney (**Fig. 5e**). Immunofluorecence for the lysosomal marker, LAMP-1, showed a marked decrease of LAMP-1 signal, especially in neurons, throughout the CNS, and in somatic tissues in rAAV9-treated MPS IIIB mice (**Fig. 5f**). These data indicate that rAAV9-mediated hNAGLU is functional and sufficient for correction of lysosomal storage pathology in both the CNS and somatic tissues.

Immunofluorecence for GFAP was performed to determine the impact of rAAV9 treatment on astrocytosis, a major hallmark secondary neuropathology of MPS IIIB [6], [10]. We observed significant decreases in the number of astrocytes in gray matter throughout the brain in treated MPS IIIB mice compared to non-treated controls (**Fig. 5g** **),** strongly indicating the amelioration of astrocytosis in MPS IIIB by the rAAV9-hNAGLU gene delivery.

### Correction of the Aberrant Molecular Pathways Associated with Neurodegeneration in the Brain and Blood

Blood and tissue analyses were carried out to determine whether the systemic rAAV9-hNAGLU gene delivery had an impact on the dysregulation of selected genes (**Table 1**) in the brain, blood, and representative control tissue (liver or lung). Using qRT-PCR, we observed that rAAV9 treatment resulted in a significant increase in the expression of all tested genes in blood to wt levels, including Necab3, Apbb2, App, Saa1, Prkcg, Park2, Snca1, and Prnp (**Fig. 2a** **,** **3a** **,** **4a**). Our data also showed normalized or near normalized expression of Necab3, Prkcg, Park2, and Prnp2, in the brain of rAAV9-treated MPS IIIB mice at 6 mo age (**Fig. 2a** **,** **3a** **,** **4a**). Importantly, rAAV9 treatment did not significantly impact on the expression of these genes in the control somatic tissue (liver or lung) (**Fig. 2a** **,** **3a** **,** **4a**). Further, IHC showed significant clearance of Necab3 (**Fig. 2b, 2c**), Snca (**Fig. 3c, 3d**), Park2 (**Fig. 3e**), and Prnp (**Fig. 4b, 4c**) in the brain and spinal cord of rAAV9-treated MPS IIIB mice. Using ELISA, we detected a marked reduction of pApp in the cortex (**Fig. 2d**) of mice receiving rAAV9-hNAGLU vector. These results demonstrate that restoration of NAGLU activity in the CNS led to the correction of neurodegeneration in MPS IIIB mice, which correlated with normalization of ostensibly CNS-specific transcripts in blood.

## Discussion

This study presents molecular evidence demonstrating that blood gene expression profiles vary with CNS disease progression in MPS IIIB mice. There is also a first indication that correction of the disease pathology by effective restoration of NAGLU activity reverses dysregulated gene expressions in the blood. The blood genome-wide gene expression array on pooled samples allowed a comprehensive view of a diverse set of pathological changes within a single set of data, providing a new strategy for investigating disease mechanisms. This is particularly important because the pathology of MPS IIIB is very complex, with abundant metabolic and immune dysfunctions secondary to the lysosomal storage. Our data showed that MPS IIIB led to the dysregulation of nearly 3,000 genes in blood, including numerous genes involved in a broad range of biological processes. Of these genes, many are considered to be tissue-specific, and not anticipated to be differentially regulated in blood. The observed blood gene dysregulation reflected the complexity of MPS IIIB pathology, with pathways representing virtually all known pathological aspects of the disease, including inflammation [5]–[10], neurodegeneration [6], [8], [10], [14]–[19], autophagy [12], [13], oxidative stress [9], [11] and metabolic (GAGs, lipid, fatty acid, cholesterol metabolism) impairments [4], [5], [18], and cellular repair processes. This suggests that peripheral blood harbor significant amounts of information linked to pathological changes in MPS IIIB, including those of the CNS. We were specifically drawn to a group of genes that were significantly dysregulated in blood and brain, including Necab3, Apbb2, App, Psen1, Saa1, Prkcg, Park2, Snca and Prnp, because of their involvement in neurodegenerative diseases, such as Alzheimer’s and Parkinson’s disease [14]–[16], [18], [20]–[23]. This supports that MPS IIIB shares important aspects of neuropathology with these diseases [14]–[18].

The observation that components of blood transcripts correlate with dysregulated CNS transcripts in MPS IIIB mice offers the possibility of a readily accessible tool to evaluate neurodegeneration associated with this disease. This is particularly relevant to the assessment of current and planned therapeutic interventions including enzyme replacement therapy, bone marrow transplant, and gene therapy. We have previously demonstrated that a trans-BBB gene delivery approach, using systemic injection of rAAV9-NAGLU vector, led to global CNS restoration of functional NAGLU, correction of lysosomal storage pathology and functional neurological benefits in adult MPS IIIB mice. [28] We further demonstrated here that rAAV9 gene delivery mediates the correction of the MPS IIIB neurodegenerative manifestations at both the molecular and cellular levels. In this study, this treatment corrected a large portion of the dysregulated genes in blood that are generally associated with CNS pathology, including amyloidosis, synucleinopathy and prion encephalopathy, at the RNA and/or protein levels in both the blood and brain. Importantly, neither disease progression, nor correction by gene delivery altered expression of these CNS-specific pathways in control somatic tissues, although lysosomal storage is evident and is widely corrected in these tissues by the systemic gene delivery treatment. This supports the idea that the changes in peripheral blood reflect conditions in the brain. In other words, blood may harbor potential CNS-specific biomarkers for MPS IIIB. Genome-wide gene expression microarray on blood may provide a useful means to screen for molecular pathway impairments and identify molecular signatures that are relevant to MPS IIIB pathology, as previously observed in patients with amyotrophic lateral scleroses (ALS)[44] and PD [45]. This is particularly important as effective therapies become available for MPS IIIB because of limited access to CNS tissues in humans.

Although neurodegeneration associated pathways were broadly anomalous in both the blood and brain of MPS IIIB mice, in general, the expression of these genes was increased in the brain but reduced in blood. While the mechanisms of this brain-blood correlation are unclear, it is known that the majority of nucleated blood cells are leukocytes. The role of these leukocytes as circulating immune cells suggests the possibility that these changes reflect the neurodegenerative status in the CNS through immune surveillance of these tissues. The observed changes in Necab3 and Apbb2 expression in blood, evident at 2 mo age, preceded alterations in these transcripts in the brain, which were not detected until 6 mo age. While the histopathology of MPS IIIB in the CNS begins at an early age with the development of astrocytosis and lysosomal storage lesions, cognitive impairments aren’t detected until 5.5–6 months of age. This suggests that the changes in these blood transcripts are early indicators of the neurodegenerative status in this mouse model.

The breadth of dysregulation of transcripts in MPS IIIB mice reflects the progressive impairment of pathways linked to neurodegeneration. While Necab3, Apbb2, App, Psen1, Prkcg and Saa1 are associated with Aβ accumulation, Park2 and Snca are associated with protein aggregation in AD and PD, as well as other neurodegenerative diseases [20]–[23]. While it is possible that the pathological changes in these pathways all contribute to the progressive cognitive decline in MPS IIIB, as Aβ accumulation is the hallmark of AD [20] and Snca is associated with dementia in PD, they may also represent downstream effects of the ongoing accumulation of GAGS in the CNS [46].

We also found evidence of prion encephalopathy in the MPS IIIB mice. As was the case with the majority of the dysregulated genes, Prnp mRNA was significantly increased in the brain but reduced in the blood of 6 mo-old MPS IIIB mice. Extracellular deposition of Prnp protein was detected in the brain and spinal cord. However, there was no significant change in Prnp mRNA in representative somatic tissue (lung). Cellular Prnp (Prnp^c^) is a small cell surface glycoprotein which is considered the physiological form of Prnp, but can be converted into the scrapie Prnp (Prnp^sc^) by protein mis-folding [47]. Prnp^sc^ is the major pathological Prnp conformer that present in high quantities in the brains of animals and humans with infectious neurodegenerative prion diseases, such as sheep scrapie, bovine spongiform encephalopathy, Creutzfeldt-Jacob disease, and Gerstmann-Straussler syndrome [48]. Numerous studies have shown evidence linking Prnp to non-prion neurological diseases, such as AD, Down’s syndrome, primary progressive aphasia, Wilson’s disease and temporal lobe epilepsy [49]. Histologically, Prnp was shown to often accompany Aβ-positive plaques in the brain of AD patients and Prnp^c^ may also mediate the toxicity of Aβ oligomers that are associated with AD [47], [50].

A common element in each of these pathologies is the accumulation of misfolded proteins, and in the case of MPS IIIB, this may be an indirect result of the progressive failure of lysosomal function. Importantly, the development of these distinctive CNS pathologies, and their measurable effects on transcription in peripheral blood, may provide an accessible tool for assessing the effects of therapies on MPS IIIB disease progression. Though the mechanisms remain unclear, we have demonstrated that the CNS-blood correlation in neurodegeneration in MPS IIIB can be corrected by systemic rAAV9-hNAGLU gene delivery, which effectively treats the CNS disorder. This supports the possibility of peripheral blood as an effective and minimally invasive tool for biomarker and disease mechanism studies, not only for MPS IIIB, but potentially other neurological diseases as well.

## Supporting Information

Table S1 **Real-time PCR Primer Sequences.**
(DOCX)
