# Supplementary material for: Amyloidosis, Synucleinopathy, and Prion Encephalopathy in a Neuropathic Lysosomal Storage Disease: The CNS-Biomarker Potential of Peripheral Blood
Source: PLoS One. 2013 Nov 21;8(11):e80142. doi: 10.1371/journal.pone.0080142 (PMC3836978; doi:10.1371/journal.pone.0080142)
Supplement: Table S1 — Real-time PCR Primer Sequences. (DOCX) [file pone.0080142.s001.docx]

| **Table S1: Real-time PCR Primer Sequences** | | | |
| --- | --- | --- | --- |
| **Gene symbol** | **Gene name** | **Primer/Probe** | **Sequence** |
| **Actb** | Beta Actin | Forward | CTGGCACCACACCTTCTACA |
|  |  | Reverse | AGGTCTCAAACATGATCTGGGT |
|  |  | Hex probe | CTGTGCTGCTCACCGAGGCC |
| **Prnp** | Prion protein | Forward | CTTCAGCTTGCACTGTGGAT |
|  |  | Reverse | AGGGTCACACGGTAAGCATT |
|  |  | Fam probe | CCCTGGCCGTTCCATCCAGT |
| **Prkcg** | Protein kinase C, gamma | Forward | ATTGCACCTCCTTTCAGACC |
|  |  | Reverse | GTAAAGCCCTGGAAATCAGC |
|  |  | Fam probe | CCGCCAGACCGCTTGGTTCT |
| **Apbb2** | Amyloid beta (A4) precursor protein-binding, family B, member 2 | Forward | CCACTTCCTCCTGGAAAGAC |
|  |  | Reverse | GCTGTCAGATCAAGGAGTCAAA |
|  |  | Fam probe | CAGTCAACAGCAAATGGCAACACAA |
| **Park2** | Parkinson disease (autosomal recessive, juvenile) 2, parkin | Forward | AAGCTCTGTGGCTCTCCAGT |
|  |  | Reverse | ACACTCACACAATGCAGGCT |
|  |  | Fam probe | TGCTCACACACTGTGGATGCCTG |
| **Necab3** | N-terminal EF-hand calcium binding protein 3 | Forward | TCTACCATCAGTGGTTGTACCAG |
|  |  | Reverse | GATAGGCAAATGACATGCAAAC |
|  |  | Fam probe | CCCACCCATTCTCAGGGTACTAAGGG |
| **Psen1** | Presenilin 1 | Forward | CGTGCTCTGCTAGCTTTGAC |
|  |  | Reverse | GCTCTGTTTGGTTCACCTCA |
|  |  | Fam probe | TGCGGGTAAATCTCCATGCCC |
| **App** | Amyloid beta (A4) precursor protein | Forward | CACATCGTGATTCCTTACCG |
|  |  | Reverse | GTCTCACAAACATCCATCCG |
|  |  | Fam probe | TCTCGTGCCCGACAAGTGCA |
| **Snca** | Synuclein, alpha | Forward | CTGGCAGTGAGGCTTATGAA |
|  |  | Reverse | TTGGGTGCAATGACATTCTT |
|  |  | Fam probe | TCTTGGTAGCCTTCCTCTGAAGGCA |
| **Saa1** | Serum amyloid A 1 | Forward | GAACACTGAAGATGCTCTCTGG |
|  |  | Reverse | CCTCTCCTCCTCAAGCAGTT |
|  |  | Fam probe | TCATGTGTGTATCCCACAAGGGTTTCA |
| Probes were labeled with either Hex or Fam. All samples were run in triplicate, as duplicate reactions with β-actin as internal control. | | | |
